# Calcium Phosphates: A Key to Next‐Generation In Vitro Bone Modeling

**DOI:** 10.1002/adhm.202401307

**Published:** 2024-08-23

**Authors:** Ashish Pandit, Abhishek Indurkar, Janis Locs, Håvard Jostein Haugen, Dagnija Loca

**Affiliations:** ^1^ Institute of Biomaterials and Bioengineering Faculty of Natural Sciences and Technology Riga Technical University Pulka Street 3 Riga LV‐1007 Latvia; ^2^ Baltic Biomaterials Centre of Excellence Headquarters at Riga Technical University Riga LV‐1007 Latvia; ^3^ Department of Biomaterials University of Oslo Oslo 0317 Norway

**Keywords:** angiogenesis, bone, calcium phosphates, in vitro bone model, mechanical strength

## Abstract

The replication of bone physiology under laboratory conditions is a prime target behind the development of in vitro bone models. The model should be robust enough to elicit an unbiased response when stimulated experimentally, giving reproducible outcomes. In vitro bone tissue generation majorly requires the availability of cellular components, the presence of factors promoting cellular proliferation and differentiation, efficient nutrient supply, and a supporting matrix for the cells to anchor – gaining predefined topology. Calcium phosphates (CaP) are difficult to ignore while considering the above requirements of a bone model. Therefore, the current review focuses on the role of CaP in developing an in vitro bone model addressing the prerequisites of bone tissue generation. Special emphasis is given to the physico‐chemical properties of CaP that promote osteogenesis, angiogenesis and provide sufficient mechanical strength for load‐bearing applications. Finally, the future course of action is discussed to ensure efficient utilization of CaP in the in vitro bone model development field.

## Graphical abstract

The review examines the need for in vitro bone models focusing on the role of calcium phosphates (CaP). It discusses CaP’s contributions to cell types, nutrient supply, and mechanical strength, while addressing challenges like compositional variability and instability. The manuscript also suggests future directions for utilizing CaP in developing effective in vitro bone models.

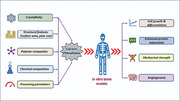

## 1 Introduction

### 1.1 Anatomy of Bone

**Figure 1:**
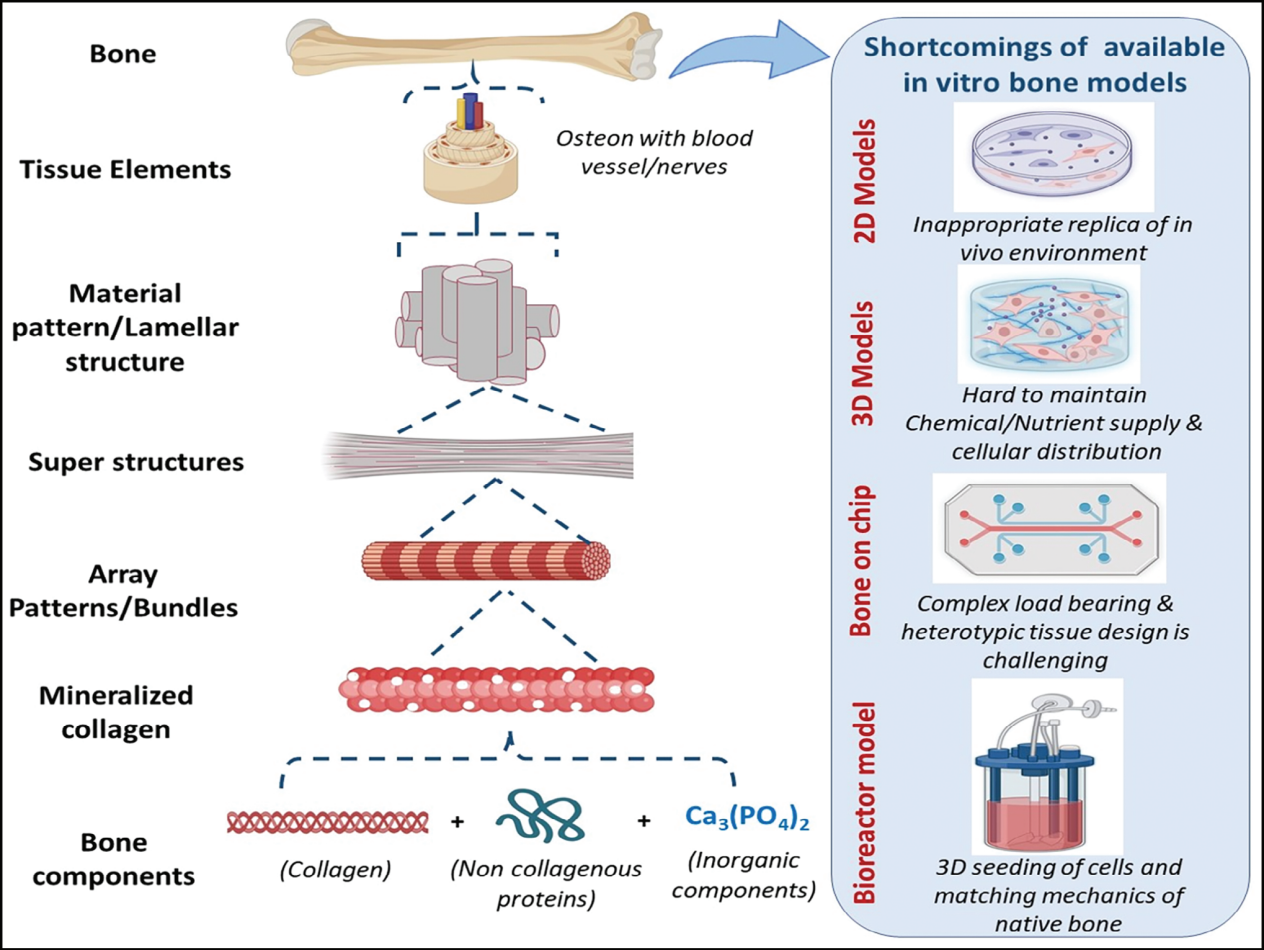
Hierarchical structure of bone and shortcomings of available in vitro bone models.

Human infants generally have 270 bones which fuse to ≈206 in adults.^[^ ^1^ ^]^ These bones provide structural support to the body, aiding in locomotion and protecting vital inner organs. They play a pivotal role in regulating calcium and phosphorus levels inside the body, acting as centers for hematopoiesis.^[^ ^2^ ^]^ Large bones follow complex arrangements with a hollow shaft – diaphysis in the middle, and growth plates sandwiched between conical‐shaped metaphysis underneath and epiphyses on the top. The diaphysis is made of dense cortical bone, whereas epiphysis and metaphysis comprise trabecular meshwork lined with cortical bone.^[^ ^3^ ^]^ Overall, the human skeleton contains 80% of cortical bones, and 20% are trabecular. Structurally, cortical bone is dense and circumvents the bone marrow, whereas trabecular bone possesses a structure resembling a honeycomb‐like lattice of trabecular rods/plates dispersed within the bone marrow. The hierarchical structure of bone as represented in **Figure** **1** is very complex, thus tricky and challenging to replicate in vitro. Several shortcomings encompassed by the existing in vitro bone models, like inadequate replication of the in vivo environment, difficulties in maintaining nutrients, cellular distribution, and challenges in complex load‐bearing applications are well reported.^[^ ^4^, ^5^, ^6^, ^7^, ^8^ ^]^ Further, the concept of calcium phosphates has already been extensively documented.^[^ ^9^, ^10^, ^11^ ^]^ Therefore, in this review, we provide an overview of the need for in vitro bone models, their requirements, and the challenges faced in current research, with a focus on calcium phosphates. Furthermore, key concepts related to the role of calcium phosphates in supporting their effective utilization are discussed, with special attention to their properties that drive cellular interactions such as osteogenesis and angiogenesis, while also providing mechanical strength.

Osteons/Harversian canals form a primary unit of cortical and trabecular bone, with dimensions of 400 nm in length and 200 nm in width at the base. These osteons branch within the cortical bone and their boundaries are formed with concentric positioning of lamellae, which contain collagen fibrils in alternating positions.^[^ ^12^ ^]^ The typical extracellular matrix in bone comprises a complex arrangement of type 1 collagen fibrils co‐assembled with noncollagenous proteins loaded with apatite crystals. Three degrees of porosity exist within the bone matrix, namely, vascular porosity within the Volkmann and Harversian canal (20 µm radius), lacunar canalicular system (0.1 µm radius) in mineralized bone tissue, and spaces inside collagen fibers and crystallites of mineral hydroxyapatite (0.01 µm radius).^[^ ^13^ ^]^ Chemically, bone comprises 70% mineral and 20% organic content, while the rest comprises water and lipidic materials, including small amounts of polysaccharides and proteins. Calcium phosphate constitutes the major inorganic component, with trace amounts of magnesium, carbonate, silica, iron, zinc, etc. Crystalline and amorphous calcium phosphate are interspersed within the collagen matrix, providing stiffness to the bone.^[^ ^12^ ^]^ Bone undergoes both lateral as well as transverse growth during early life. After that, bone tissue undergoes continuous remodeling for homeostasis and mending the damaged bone. Under normal conditions, bone remodeling comprises four sequential steps: acquisition and stimulation of mononuclear progenitor cells, resorption action of mineralized and organic matrix by the osteoclasts, and restoration of the resorbed surface by osteoblastic deposition activity. Osteoblasts, osteoclasts, and various other cell types contributing to normal functioning of the bone are described in forthcoming sections.^[^ ^14^ ^]^

### 1.2 Need for In Vitro Bone Models

Statistically, nearly one‐third of hospitalized individuals in Europe are affected with bone‐related injuries or disorders where therapeutic interventions incur tremendous cost.^[^ ^15^ ^]^ The injury's extent, along with individual factors like age, gender, lifestyle (physical activity, diet, etc.), and genetics, highly affect bone healing. The combined effect of such factors compromises bone health, causing disorders like osteoarthritis and osteoporosis. Higher life expectancy, coupled with the increasing geriatric population, has caused a paradigm shift in focus toward management of bone disorders. Anatomical regions like the hip, spine, and wrist are prone to frequent fractures in bone disorders like osteoporosis. Globally, 1.6 million hip fractures are reported due to osteoporosis, and by 2050, this number is estimated to be ≈6.3 million.^[^ ^16^ ^]^ Growing orthopedic injuries and surging cases of bone diseases have translated to higher demands for bone implants. Their market is estimated to grow with a compound annual growth rate (CAGR) of 5.7% from 2023 to 2028, with a total worth of ≈73 billion USD by 2028.^[^ ^17^ ^]^ Undoubtedly, the numbers correlate with increasing demand for better treatment options which propel orthopedic research.

Animal models remain a mainstay in orthopedic investigations. Although preferred, inconsistencies in reporting the bone phenotype of animal models often complicate the interpretation of results and comparison of studies. Moreover, animal models lead to economic constraints, thereby strengthening the candidature of alternative approaches.^[^ ^18^ ^]^ The excellent load‐bearing and mechanical strength provided by the stratified positioning of calcium phosphate minerals within the collagen matrix has garnered significant attention from material scientists to replicate this system in vitro. Specifying pertinent animal models for bone diseases, genetic mutations, and high economic input are some other challenging factors for the application of animal models in studying human bone. Moreover, preclinical studies often do not translate into successful clinical trials. Additionally, differences in human and animal physiology limit the adaptation of potential therapies.^[^ ^19^ ^]^ To overcome these drawbacks, analyzing bone disorders and testing of therapeutics, in vitro models are looked after.

Promoting science and innovation while harmonizing legal requirements with the use of animals in research is cumbersome. This would only be achieved by adopting transparency in the experimental methodology. In order, to abolish discrepancies in regulations followed by members of the European Union (EU) regarding the protection of animals for scientific purposes in 1986, the directive 86/609/EEC was enforced. The principal intent of this directive was to confirm that animals utilized for investigational purposes are cared for and not subjected to unwanted pain or suffering. After that, in 2010, directive 2010/63/EU was adopted to coordinate animal research legislation across member states, guaranteeing high levels of animal welfare in scientific pursuits. The directive highlights to achieve the necessity to execute the 3R principles of Replacement (replacement of experimental animals with alternative methods), Reduction (reducing the total count of animals employed in investigation to achieve valid results), and Refinement (refining current methodologies to reduce pain and suffering endured by animals). Research entities must obtain prior approval from the animal ethics committee as described in Annexure VI of the directive 2010/63/EU, justifying the 3 “R” principles. According to Directive 2010/63/EU (Article 54(1)) as amended by Regulation (EU) 2019/1010, member states are required to submit a report on the enactment of the above laws by 10 November 2023 and periodically after every 5 years. Similarly, the passing of legislation in the US making animal experiments nonmandatory for new drugs to secure FDA approval is encouraging the pursuit of in vitro models.^[^ ^20^, ^21^, ^22^ ^]^

## 2 Requirements of In Vitro Bone Models

Bone tissue generation in vitro majorly requires the availability of four components, i.e., cells capable of differentiating into osteoblasts, presence of inductive factors that promote cell proliferation, efficient supply of nutrients, and supporting matrix for the cells to anchor and gain defined topology.^[^ ^23^, ^24^ ^]^ Countless investigations in the scientific domain discuss the characteristics of the aforementioned components. However, these factors are discussed in detail in the forthcoming section, emphasizing their possible shortcomings that need to be overcome.

### 2.1 Cells

Bone comprises four different cell types: osteoblasts, osteocytes, osteoclasts, and bone lining cells. Bone tissue engineering concepts have been employed to generate in vitro bone models as possible replacements for in vivo models.^[^ ^25^ ^]^ Concomitant deployment of osteogenic and osteoclastic cells with endothelial cells is advantageous to study bone models. Hematopoietic cells and vessel‐forming endothelial cells alter bone metabolism; hence, their inclusion while developing co‐culture for in vitro models is imperative.^[^ ^26^ ^]^ With the provision to co‐culture only limited cell categories, the purpose of in vitro models must be clearly outlined. Osteocytes constitute 95% of the cellular component in any human adult bone and drive most of the bone functions. Transduction of mechanical signals while facilitating endocrinal regulation and bone remodeling are a few key responsibilities associated with osteocytes. Human osteocytes are sourced from the digestion of trabecular bone in presence of chelating agent and collagenase enzyme. Bone marrow stromal cells (BMSCs) withhold properties to differentiate into osteoclast and osteoblast cell types. Interestingly, undifferentiated BMSCs facilitate vascular network formation upon deployment with endothelial cells.^[^ ^27^, ^28^ ^]^

Endothelial cell (EC) lines, namely Human umbilical vein endothelial cells (HUVEC), Human Lung Microvascular Endothelial Cells (HMVEC), and Human endothelial progenitor cells (EPCs) either alone or in combination, are routinely deployed for initiation of vascularization while developing in vitro models. It is challenging to source endothelial cells from human origin. Further, parathyroid hormone (PTH) and estrogen are some of key drivers in bone angiogenesis. Therefore, shortlisted cells should be responsive to these hormones. Additionally, different EC growth (EGM) media EGM‐1 and EGM‐2 are usually deployed to expedite vascularization. Typically, EGM‐2 is low serum media augmented with several angiogenic factors like vascular endothelial growth factor (VEGF), hydrocortisone, epidermal growth factor (EGF), insulin like growth factor (IGF), ascorbic acid (AA). Literature reviews outlining different cellular phenotypes for in vitro bone modelling are available in scientific domain.^[^ ^29^, ^30^, ^31^ ^]^

### 2.2 Modulators of In Vitro Bone Models

Osteocytes undergoing apoptosis secrete molecules that potentiate resorption activity by osteoclasts.^[^ ^32^ ^]^ For instance, e.g., interleukin‐6, Macrophage stimulating factor (M‐CSF) moderate osteoclast activity and generation. M‐CSF, RANKL (Receptor Activator of Nuclear Factor kappa‐Β Ligand), and Osteoprotegerin (OPG) can guide bone modeling.^[^ ^33^ ^]^ The culture media employed should be infused with the factors above depending on the growing cell type(s). Supplementation of osteogenic media with dexamethasone, β‐glycerolphosphate (β‐GP), and ascorbic acid is routinely followed for inciting proliferation and differentiation into osteoblasts, causing mineral deposits.^[^ ^34^ ^]^ Glucocorticoids like dexamethasone (Dex) support the differentiation of bone marrow cells into an osteoblastic lineage that later develops bone‐like tissue outside the cell. However, specific results have demonstrated that Dex treatment is not an ultimate parameter determining bone formation on CaP materials like hydroxyapatite (HAp).^[^ ^35^ ^]^ Ascorbic acid is an essential co‐factor involved in collagen synthesis.^[^ ^36^ ^]^ Infusion of agents above is predominantly associated with osteoblast differentiation. Still, these are also closely related to osteoclast activity. Primarily, the osteoclastogenic culture medium is complemented with RANKL and M‐CSF. At the same time, other agents utilized in vitro to boost bone formation are parathyroid hormone (PTH), Vitamin D, VEGF, and collagen.^[^ ^37^ ^]^ Lastly, growth factors like the Basic fibroblast growth factor (bFGF) promote osteogenic differentiation of MSCs and the introduction of bone morphogenic protein‐2 (BMP‐2) in vitro leads to enhanced proliferation of osteoblasts.^[^ ^38^, ^39^ ^]^ Apart from media supplements, dissolved gases, and mechanical stress positively affect bone formation. Intermittent compressive force (ICF) that is analogous to mechanical stress applied caused a marginal increment of 13% in the tension of Oxygen (O_2_) and Carbon dioxide (CO_2_) inside an in vitro chamber, categorically regulating mineralization activity by the cells.^[^ ^40^ ^]^

### 2.3 Nutrient Supply and Angiogenesis

Vascularization is pivotal in endocrinal ossification – a process where bone grows and regenerates.^[^ ^41^ ^]^ Vascular ingrowth facilitates the supply of progenitor cells and nutrients critical to several bone operations, including regeneration and metamorphosis of soft cartilaginous tissue into mineralized one.^[^ ^42^ ^]^ Osteogenesis is always preceded by vascularization in bone tissue formation.^[^ ^43^ ^]^ In vitro bone constructs are susceptible to diffusion issues hence the presence of an operational angiogenic system could be a pre‐requisite. It can be introduced into the bone construct either by top‐down or bottom‐up approaches. In the top‐down approach, luminal surfaces are drawn with a micro‐physiological device, followed by its coating with endothelial cells. Contrarily, the self‐assembly of endothelial cells is targeted in the bottom‐up method under the supervision of angiogenic factors like VEGF, Fibroblast growth factors (FGF), Platelet‐derived growth factor (PDGF), etc. This pathway, being analogous to the physiological state, is highly endorsed.^[^ ^31^ ^]^ However, small molecules (like VEGF) have short half‐life and, when introduced into the model, require optimization of release kinetics, compelling introduction of a multifunctional carrier like CaP that would sustain VEGF release while possessing the angiogenic potential of its own.

### 2.4 Mechanical Strength

In terms of mechanical strength, cortical bone exhibits anisotropy with greater strength along the longitudinal axis as against radial and circumferential directions. Elastic modulus of human femoral cortical bone is ≈17 900 ± 3900, whereas it is 1.7 times lower in transverse direction. Trabecular bone being more porous than cortical bone also displays anisotropic mechanical properties dictated by its porosity.^[^ ^4^ ^]^ The primary mineral component of bone is calcium phosphate, which, alongside nonmineralized components like collagen, maintains the mechanical integrity of the structure.^[^ ^44^ ^]^ Cells in bone tissue undergo diverse mechanical stresses and compressional forces, which drive their functions. Consequently, the 3‐dimensional (3D) arrangement in scaffold‐based in vitro system aims to replicate structural, mechanical, and regulatory cues of natural extracellular matrix. Perfusion bioreactors subject the cells contained in scaffolds to a tightly controlled environment, enabling mechanical stimulation through shear stress. This helps regulate both biochemical and mechanical signals during cell culture. Replication of bone in a lab environment therefore demands a 3D organization to mimic native structure and calls for the involvement of scaffolds capable to withstand the subjected mechanical conditions.^[^ ^45^ ^]^ Advances in the bone tissue engineering have resulted in the deployment of numerous polymers, ceramics and metal alloys (titanium, cobalt‐chromium alloys) for bone formation.^[^ ^46^ ^]^ Polymeric materials exert proper control over characteristics such as size and porosity of the in vitro bone construct. Synthetic polymers like poly (lactic‐acid) (PLA), poly(glycolic‐acid) (PGA), poly(caprolactone) (PCL), and their copolymers are at the forefront for bone formation owing to their higher mechanical strength. Nevertheless, the major shortcoming of synthetic polymers is inadequate cellular adhesion ranging from 2% to 8% for Mesenchymal Stem Cells (MSC) and chondrocytes if such polymers as PCL, PLA, and poly (lactic acid: polyglycolic acid = 75:25) (PLGA) were used. However, surface coating with arginine could enhance it to values greater than 25%.^[^ ^47^, ^48^ ^]^ On the other hand, natural polymers like collagen, chitosan, cellulose, silk, etc., are inherently hydrophilic and readily undergo chemical modulation to suit the requirements of in vitro bone models. For instance, chitosan is widely deployed for bone tissue formation; however, it lacks adequate mechanical strength but allows preferential attachment and proliferation of osteoblasts. So, to strike a balance and achieve adequate mechanical properties with higher osteoconduction ability, composite scaffolds of PLA and HA with chitosan were fabricated by 3D printing.^[^ ^49^, ^50^ ^]^ There is a pressing need to effectively utilize components from different origins and build feasible in vitro bone models.

## 3 Role and Effective Utilization of Calcium Phosphates

Effectively utilization of calcium phosphates (CaP) to satisfy the requirements of in vitro bone models as mentioned in section 3 is crucial. However, there are certain physicochemical and mechanical properties of CaP which need comprehensive understanding. Modulation of such properties determine cellular interaction of CaP material under consideration. Therefore, in forthcoming subsections, important physicochemical and mechanical properties of CaP are discussed with relevance to their impact on biological properties.

### 3.1 Surface and Physicochemical Properties

Surface and physicochemical properties control the behavior of Calcium phosphate (CaP) ceramics. Properties like porosity and density of obtained bioceramics are highly dependent on its sintering temperature and other sintering process parameters.^[^ ^51^ ^]^ CaPs generated with high porosity and rugged surfaces impart higher surface area with an optimum 3D environment promoting osteoinduction.^[^ ^52^ ^]^ The typical surface area for osteoinductive materials lies in the range of 0.7–1.6 m^2^ g^−1^, whereas in the case of noninductive CaP ceramics, it is manifold lower.^[^ ^83^, ^109^ ^]^ In some instances, the native calcium phosphate ceramics like β‐tricalcium phosphate (β‐TCP) (Osferion, surface area: 4 m^2^ g^−1^) and Hydroxyapatite (HAp) (surface area: 1–2 m^2^ g^−1^) were reprecipitated to corresponding derivatives with higher surface areas of 22.5 and 30 m^2^ g^−1^, respectively. Transformed β‐TCP and (HAp) exhibited enhanced osteoinductive properties compared to their native counterparts.^[^ ^53^ ^]^

Further, sintering greatly impacts the density, porosity, grain size, and crystallinity of the CaP ceramics.^[^ ^54^ ^]^ Particularly in the case of HAp, the linear shrinkage exhibited an upward trend, rising from 22.2% at 1000 °C to 30.2% at 1450 °C with a maximum shrinkage of 30.8% observed at 1250 °C. Porosity was inversely related to shrinkage value as minimal porosity (<1%) was noted at 1250 °C. Subsequently, a proportional increment in mean grain size was observed in samples with elevation of sintering temperature. HAp specimens sintered at temperatures of 1250 and 1400 °C displayed the mean grain size of 2.03 and 12.26 mm, respectively.^[^ ^55^ ^]^ Calcium phosphate embedded on the glass plate by sol–gel method and treated at different ranges of temperatures generated HAp capable of anchoring murine bone marrow cells on its surface in vitro. Elevation in sintering temperature from 400 to 1000 °C caused a concomitant increment in crystallinity and grain size of the developed HAp while sintering temperatures of ≈1000 °C induced the formation of specific topography in HAp layers favoring the formation of the biological matrix. The obtained results indicated that collagen produced by the cells fused with the HAp was indistinguishable from underlying glass/quartz support. These in vitro studies have attested to the importance of HAp in promoting bone growth, thus strengthening its candidature toward studying bone bonding in case of fractures.^[^ ^56^ ^]^

**Figure 2:**
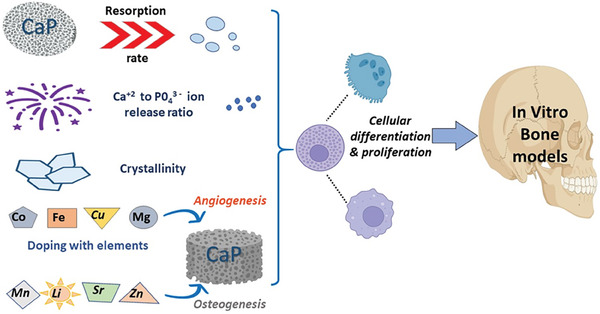
Calcium phosphates and their physicochemical properties favoring in vitro bone formation.

Surface geometry and internal porous structure also hugely impact the osteoinductive properties of CaP structures with connected macropores over scale of 300–500 µm are preferable as they allow exchange of metabolites, by aiding osteogenesis also promoting neo‐vasculature.^[^ ^57^ ^]^ Similarly, micropores (diameter <50 µm mostly ≈2–10 µm) enhance protein adsorption, calcium (Ca^2+^) and phosphate (P0_4_^3−^) ion exchange thereby contributing toward mineralized cellular depositions.^[^ ^58^ ^]^ This phenomenon explains osteoinductivity by the microporous structure of HAp scaffolds. CaP surface topology also affects the cellular response to the extent that flat surfaces ineffectively contribute toward cellular adhesion while rough surfaces amplify cellular interactions. Contrary to concave hydroxy apatite, convex surfaces promoted fluid flow and cellular migration in canine models whereas, contradictory outcome was noted for concave morphology in rectus abdominis muscle of baboon model. Under normal physiological condition, osteoclasts secrete acids causing bone resorption in localized areas, thereby creating micropores with near‐circular geometry. Similarly, the surface topology of the HAp construct influences the pace of bone regeneration. Incubation of MC3T3‐E1 cells on the HAp plates (2 mm thickness) having semi‐circular channels and circular pores for 4 weeks depicted faster filling of circular pores due to their closer resemblance to the in vivo conditions.^[^ ^59^, ^60^, ^61^ ^]^ **Figure** **2** illustrates the physicochemical properties calcium phosphates favoring in vitro bone formation.

**Figure 3:**
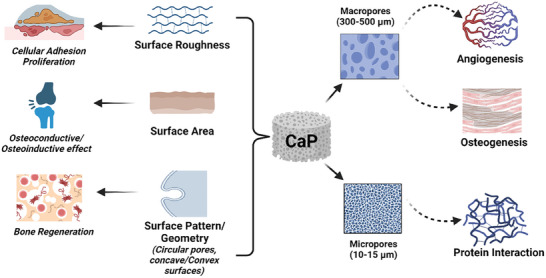
Topological characteristics of calcium phosphates promoting bone formation in vitro.

Besides surface geometry, the adsorption of proteins from the biological milieu onto the surface of calcium phosphates plays a significant role in governing cell adhesion and guiding various cellular processes, including proliferation and differentiation. This, in turn, drives the end applications of calcium phosphates. The relative efficiency of osteoinductive β‐TCP (sintering temperature: 1100 °C) and nonosteoinductive HAp (sintering temperature: 1250 °C for 8 h) for adsorption of serum proteins was evaluated in vitro. Briefly, β‐TCP and HAp particles of 2–3 mm diameter were immersed into 3 mL of osteogenic medium, and the extent of protein adsorption was analyzed. In vitro analysis with Fetal bovine serum (FBS) concluded that Alpha‐2‐HS‐glycoprotein (Fetuin A) possessed 17 times higher affinity for β‐TCP. In contrast, such proteins like Matrix gla protein and Cytochrome C were adsorbed to a value of 0.7 times higher as compared to the HAp surface. HAp predominantly attracted matrix Gla protein (MGP) up to 1.8‐folds greater than β‐TCP at the end of 48 h. Eventually, mesenchymal stem cells (hMSCs) cultured on the surface of these two ceramic particles (3 particles each; diameter: 2–3 mm) resulted in the expression of diverse protein profiles. During the period of study, osteopontin (OPN) and bone sialoprotein I (BSP‐1) were greatly deposited on β‐TCP. From the protein profiling, it was evident that β‐TCP rapidly supported the development of extracellular matrix if compared to HAp. At the same time, components involved in the translation and production of proteins were more prominent on the HAp surface.^[^ ^62^ ^]^ β‐TCP possessed 12 times higher surface area as against HAp and hence adsorbed proteins, which attest to its osteoinductive behavior. Conversely, it is safe to state that the type of the protein adsorbed is governed by the surface character of the applied ceramic, determining its future application. **Figure** **3** summarizes the topological characteristics of calcium phosphates and their significance in promoting in vitro bone formation.

### 3.2 CaP Chemistry

During the dissolution process, CaP bioceramics release calcium Ca^2+^ and P0_4_^3−^ in the surrounding milieu, promoting osteogenesis.^[^ ^63^ ^]^ Optimum release of P0_4_^3−^ from calcium phosphate materials favors enhanced RANKL‐RANK binding, leading to heightened NF‐ κB (Nuclear factor kappa B) signaling and robust osteoclast differentiation and further promoting in vivo bone modeling. Importantly, excessive release of P0_4_^3−^ ions is reported to reverse this effect and cause toxicity.^[^ ^64^, ^65^, ^66^ ^]^ It is well known that CaP ceramics are characterized w1 by different Ca/P molar ratios ranging from 0.5 for brushite to 1.67 for hydroxyapatite. The Ca/P molar ratio drives the biological performance of the CaP used. It has been reported that CaP ceramics with Ca/P molar ratio of 1.59 (HAp: 53%, α‐TCP: 21%, β‐TCP: 26%) significantly improved proliferation and expression of extracellular matrix genes like α‐smooth muscle actin and α‐fibronectin when compared to pure HAp (Ca/P 1.67).^[^ ^67^ ^]^ Thus, the chemistry of CaP is of utmost importance and should be carefully deliberated, considering that different CaP phases are characterized by diverse Ca/P molar ratios exhibiting varying calcium (Ca^2+^) to phosphate (P0_4_^3−^) ion release ratios which, directly influence their in vitro performance. Moreover, the calcium‐to‐phosphorous (Ca‐P) ion release ratio in the surrounding milieu dictates whether the used CaP will possess osteoinductive or osteoconductive properties. Investigations were performed with Biphasic Calcium Phosphate (BCP), β‐TCP, and HAp, synthesized using the hydrogen peroxide method and machined into plates measuring 12 mm × 2 mm for in vitro evaluation and 5 mm × 2 mm for in vivo evaluation. Calcium (Ca^2+^) to phosphate (P0_4_^3−^) ion release ratio for different ceramics like BCP (60% HAp and 40% β‐TCP), β‐TCP, and HAp were determined by Inductively Coupled Plasma Optical Emission Spectrometer (ICP‐OES) analysis post immersion of the bioceramics in deionized water for 7 days at 37 °C. Studies revealed that calcium to phosphate ion release ratios were 1.710 ± 0.125 for β‐TCP, 1.217 ± 0.087 for BCP, and 1.061 ± 0.021 for HAp. Further, osteoconductivity of HAp, BCP, and β‐TCP was examined through in vitro and in vivo osteoblastic differentiation as well as calvarial defect repair. In contrast, osteoinductivity was evaluated using pluripotent mesenchymal stem cells in vitro and observing heterotrophic ossification in muscles of murine models. BCP demonstrated higher expression of OCN, BSP, RUNX2, and Col1a genes compared to β‐TCP and HAp in osteoblasts cells, indicating higher osteoconductivity. In contrast, in the case of BMSCs, β‐TCP upregulated the same set of genes among other ceramics pointing toward an osteoinductive effect. BCP effectively repaired the rat calvarial defect, demonstrating osteoconductivity, whereas β‐TCP proved superior osteoinduction via heterotrophic ossification in rat muscles, suggesting that calcium phosphates with calcium to phosphorous release ratios in the range of 1.5–2 are more osteoinductive whereas, those in the window of 1.0–1.5 favor osteoconduction.^[^ ^68^ ^]^ This observation proves the higher osteoinductive potential of BCP (Ca/P molar ratio 1.6, sintering at 1100 °C for 8 h; 38% w/w β‐TCP) in canine in vivo model compared to HAp (Ca/P molar ratio 1.67, sintering at 1150 °C for 3 h). β‐TCP withholds greater solubility in aqueous media (which corresponds to a higher release of calcium and phosphorous ions), and this correlates with the higher osteoinductive potential of BCP.^[^ ^69^ ^]^ Similarly, amorphous calcium phosphate (ACP), owing to comparatively higher solubility, shifts cellular functions and tissue differentiation patterns by increasing local calcium ion concentrations via modulation of connexin 43 mediated gap junctions in 3D dental pulp cell (DPC) construct.^[^ ^70^ ^]^ Human osteogenic cells are proven to phagocytose calcium phosphates (β‐TCP and HAp) in vitro. The resulting elevation of intracellular calcium (Ca^2+^) levels promotes the uptake of leucine, uridine, and proline from the surrounding media. Ca^2+^ ions are postulated to play an important role in mRNA transcription and protein synthesis. Therefore, it would be safe to state that phagocytosis of calcium phosphates plays an important role in cellular differentiation.^[^ ^71^ ^]^ The osteoinductive effect of CaPs mainly attracts osteoblasts, initiating new bone formation via mineralization. This subsequently leads to the increase in the activity of the ALP enzyme, releasing free phosphate in the vicinity, causing induction of osteopontin ribonucleic acid (RNA) and associated proteins, thereby playing a major role in bone formation.^[^ ^72^ ^]^ CaPs release Ca^2+^ and P0_4_^3−^ ions, which further modulate the activation of the macrophages. These activated macrophages are known to promote bone formation.^[^ ^73^, ^74^ ^]^ Among activated macrophage phenotypes, M_1_ is proinflammatory, whereas M_2_ is anti‐inflammatory in nature. These activated macrophages can differentiate into osteoclasts and secrete various cytokines and chemokines influencing bone formation and resorption as well.^[^ ^75^ ^]^ Local inflammatory response by host tissue toward an implant causes macrophage recruitment. CaP from the implant can induce macrophage polarization and provide outcomes characterized by the chemistry of CaPs. For instance, when murine RAW 264.7 cells (with a density of 2 × 10^5^ cells per sample) were exposed to BCP (HAp/β‐TCP = 20/80) and β‐TCP (14 mm × 2 mm discs, both sintered at 1100 °C, 3 h), BCP caused preferential polarization of macrophages toward M_2_ phenotype. Similar results were obtained for the in vivo studies with murine models wherein enhanced bone formation was noted at nonosseous sites with deployment of BCP compared to β‐TCP. It was suggested that the major difference in osteoinduction might be due to the polarized M_2_ macrophages, which are pro‐osteogenic and noninflammatory in nature.^[^ ^76^, ^77^ ^]^

Inorganic components of bone tissue like calcium and phosphorus influence the mass and mineral density of bone. Furthermore, trace metal elements play a crucial role in bone mineralization and metabolism, with manganese (Mn), copper (Cu), iron (Fe), and zinc (Zn) exerting the most significant impact on bone growth and development. Substantial experimental evidence is furnished stating positive outcomes of such minerals in arresting degradation, enhancing mechanical endurance, and biological responses of CaPs. Substitution of calcium ions with elements like zinc (Zn), strontium (Sr), lithium (Li), and manganese (Mn) is reported to improve osteogenesis, whereas substitution with copper (Cu), magnesium (Mg), and cobalt (Co) magnify vascularization capability of the CaP materials. Likewise, incorporating fluorine imparts calcium phosphate crystallinity, thereby decreasing the resorption of CaPs.^[^ ^78^, ^79^ ^]^ Also, the surface charge is vital for controlling cell adhesion and signaling. Modulations in surface charge could be achieved by doping of CaPs. For example, the introduction of silicon (0.1 mol Si^4+^) in the solution combustion synthesis (SCS) technique yielded amorphous calcium phosphate with 17% and 12% by weight of β‐TCP and HAp. Escalation in negative charge from −20 to −27 mV was attained with Si^4+^ doping, causing higher proliferation and adhesion of MG‐63 cells compared to undoped samples.^[^ ^80^, ^81^ ^]^ Therefore, leveraging the potential of doped calcium phosphates for in vitro bone models is indispensable for surpassing the shortcomings of ceramic materials. Likewise, β‐TCP is favored among the calcium phosphates, for its biological properties, but susceptibility to fracture and faster resorption kinetics impair its application horizons. Hence, bioceramic materials containing Mg and Si are gaining importance for improving these properties. Comparative investigation of cultured human bone marrow derived mesenchymal stem cells (hMBSc) on akermanite (Ca_2_MgSi_2_O_7_) and β‐TCP demonstrated enhanced capability of the former to promote osteogenic differentiation as marked by enhanced expression of osteomarker genes like ALP, osteocalcin (OCN) and osteopontin (OPN). In vitro results were further supplemented by histomorphological evaluations in vivo with rabbit femur defect models, wherein it was shown that alermanite bolstered osteogenesis.^[^ ^82^ ^]^

### 3.3 Role of Calcium Phosphates in Nutrient Supply and Angiogenesis

The synergistic interplay between osteogenic precursor cells and endothelial cells is a prerequisite to empowering synchronized vascularization and mineralized tissue formation under a physiological environment.^[^ ^83^ ^]^ Ambiguity exists on the impact of endothelial cell population in biochemical pathways leading to osteogenesis. Few reports suggest favorable effects of endothelial cells on mineral deposition, whereas additional findings advocate their restrictive effect on osteogenesis.^[^ ^84^, ^85^, ^86^, ^87^, ^88^ ^]^ Therefore, induction of neovascularization and mineralization of associated tissue in vitro is strenuous. A possible bypass for this conflict would be seeding osteogenic cells before introducing endothelial cells in a scaffold‐based approach, ensuring that mineralization does not hamper vascularization.^[^ ^83^ ^]^

Calcium‐based ceramics back angiogenesis improves the transport of cells related to osteoblastic lineage, promoting bone formation.^[^ ^89^ ^]^ CaPs undergo resorption via concerted effects of dissolution and cell or protein‐based interactions.^[^ ^63^ ^]^ This enables them to modulate extracellular concentrations of Ca^2+^ and P0_4_^3−^. These ions sensed by the cells via Ras/Ref/ERK dependent pathway or adenosine governed mechanism to exercise control over cellular functions.^[^ ^90^, ^91^ ^]^ Bone injury or remodeling causes a localized inflammation, consequently leading to angiogenesis. Blood vessels promote the rapid influx of polymorphonuclear neutrophils (PMNs) and the evolution of monocytes to macrophages.^[^ ^92^, ^93^ ^]^ Activated macrophage M_1_ usually secrete pro‐inflammatory cytokines (TNF‐α, IL‐β), leading to fibrous tissue formation, while M_2_ macrophage releases anti‐inflammatory cytokines (IL‐10, TGF‐β).^[^ ^94^ ^]^ A transition is noted from M_1_ to M_2_ during the natural healing process of bone; this polarization is involved in osteogenesis. The M_2_ phenotype withholds the capacity to elicit higher angiogenic potential in vivo via secretion of VEGF, forming new blood vessels that enhance the supply of nutrients and osteoblasts precursors to the site. As mentioned earlier, calcium phosphate ceramics possess an inherent tendency toward polarization of macrophages to M_2_ type. These macrophages secrete cytokines like Interleukin‐10 and TGF‐β, thereby promoting angiogenesis.^[^ ^95^, ^96^, ^97^, ^98^, ^99^, ^100^ ^]^ However, chemical composition and surface topology are prominently involved in regulating the transformation of macrophages. BCP (±30% HAp/70% β‐TCP), possessing needle‐like morphology, and granular β‐TCP, possessing identical dimensions (1–2 mm), were tested for their activity against CD14+ monocytes in vitro. By the chemical composition and morphology BCP exhibited better polarization ability of the macrophages toward the M_2_ phenotype than β‐TCP.^[^ ^73^ ^]^ Deployment of calcium phosphate ceramics like β‐TCP, HAp, and BCP as bone substitutes in murine models revealed that porous hydroxyapatite and BCP augmented the maturation of osteoblasts prominently as compared to β‐TCP. Further, parallel investigations have discovered these maturated osteoblasts' erythropoietin and VEGF secretion capability. These molecules act on the endothelial cells to promote angiogenesis in bone.^[^ ^101^, ^102^, ^103^ ^]^ Additionally, copper‐substituted octacalcium phosphate (Cu‐OCP) was reported to promote endothelial tube formation of HUVECs in vitro. In addition to angiogenesis, collateral bone regeneration in rat calvarial models was reported with Cu‐OCP gelatin composites.^[^ ^104^ ^]^ Therefore, it can be concluded that calcium phosphates have an angiogenic potential of their own, which can be further enhanced by doping with other elements.^[^ ^42^, ^105^ ^]^ Further, adequate ligands must be represented to support vascular growth and osteogenesis. Integrins like αvβ_3_ and α_5_β_1_ are known to establish lumen in 3D, whereas αvβ_5_ and α_5_β_1_ assist osteogenesis.^[^ ^106^, ^107^, ^108^ ^]^ Considering various approaches for constructing bone in vitro, it is imperative to utilize the angiogenic potential of CaP ceramics to the fullest.

**Figure 4:**
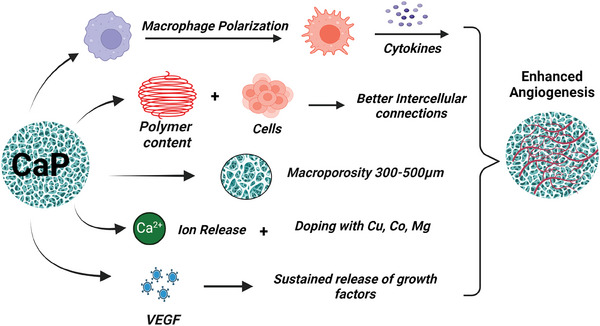
Impact of calcium phosphates on angiogenesis.

Promoting the growth of blood vessels always poses a challenge in engineered models. The introduction of growth factors, material surface modulation, and cell culture‐based techniques is a certain means of achieving the goal. Hydrogels are most often reported for delivering growth factors like VEGF.^[^ ^109^ ^]^ However, engaging calcium phosphates as multifunctional materials that would provide encapsulation and temporal release of growth factors like VEGF, while the carrier itself being osteoinductive, would prove significant for bone regeneration, concomitantly achieving angiogenesis and osteogenesis.^[^ ^105^ ^]^ Numerous reports are available wherein, mesoporous HAp microparticles and BCP scaffolds are deployed for delivery of VEGF.^[^ ^110^, ^111^ ^]^ Investigations performed with VEGF, BCP (Ca/P = 1.52 and HAp/β‐TCP range from 11/88 to 17/83), and VEGF loaded BCP on rat aortic endothelial cells in vitro demonstrated impact of BCP on osteogenesis and angiogenesis. Combining VEGF with BCP expedited upregulation of angiogenesis and osteogenesis gene expression compared to individual treatments.^[^ ^112^ ^]^ Pre‐vascularization is another encouraging alternative to using growth factors in the case of in vitro bone models. In this strategy, the Endothelial Cells (EC) are co‐cultured with mesenchymal stromal cells (MSC) inside 3D calcium phosphate structures like β‐TCP, encouraging both angiogenesis and osteogenesis.^[^ ^113^, ^114^ ^]^ Moreover, the inclusion of inorganic elements like copper and cobalt is well known to bolster the angiogenic capabilities of CaP materials associated with them. The creation of an in vitro hypoxic environment further assists blood vessel formation. This might be the mode of action for cobalt, which plays the role of hypoxia mimicker, increasing the synthesis of hypoxia‐inducible factor (HIF)−1α. Small heterocyclic compounds like phenanthroline, when dispersed in Matrigel, also create localized hypoxia‐enhancing vasculature in the murine model.^[^ ^89^ ^]^ Apart from introducing inorganic elements like cobalt and copper, polymeric content (e.g., PLA and PCL) in CaP composites could be beneficial to regulate angiogenesis. As calcium ions promote angiogenesis, sustained release of Ca^2+^ from CaP would be of great importance and could be achieved by CaP composite formation with appropriate polymer. In vitro interactions of Human umbilical vein endothelial cells (HUVEC) with calcium deficient hydroxyapatite powder – polycaprolactone (PCL) discs (10.7 mm diameter, 90 mm^2^ surface area) containing 24% PCL helped to achieve the marked generation of pre‐vascular structures compared to a composite containing 11% PCL by weight. This phenomenon might be due to the uniform distribution of HUVEC throughout the composite, which facilitates the formation of pre‐vascular structures via intercellular signaling.^[^ ^115^, ^116^, ^117^ ^]^ **Figure** **4** represents a schematic illustration of the role played by CaP in angiogenesis. The preceding strategies discussed must be explored for optimal induction of angiogenesis while building an in vitro bone model.

### 3.4 Mechanical Strength

Mechanical forces can be recognized and relayed through the extracellular matrix of bone via several elements like primary cilia on osteocytes and osteoblasts‐associated integrins. Osteoblast maturation involves the interplay of mechanical stimuli and transcriptional regulation via Runx2 and Wnt pathways.

Shear stress is generated by interstitial fluid flow inside the intricate network of bone. In vitro evaluations have proven that osteogenic cells react to this interstitial fluid shear stress (FSS) by secreting signaling factors like prostaglandins and nitric oxide.^[^ ^118^, ^119^, ^120^, ^121^ ^]^ FSS positively impacts the generation of apatite crystals in an orderly arrangement under 1 megapascal (MPa) 2‐dimensional pressure. Mechanical stimulation can also result in spontaneous rearrangement of the osteoblasts, promoting secretion of organized collagen bundles.^[^ ^122^ ^]^ Several in vitro investigations using perfusion flow have reported the effect of mechanical stress on the osteocytes derived from murine and chicken models. Shear stress (0.05–2 Pa) depicted the release of prostaglandins and nitric oxide, with concomitant uptake of Ca^2+^ inside the cells and downregulation of mRNA coding for RANKL/OPG.^[^ ^119^ ^]^ The mechnostat theory put forward by Harold Frost in 1960 postulates the level of strain experienced by the cells to be in the range of 0.03–0.15%. Below this range, bone resorption is initiated, whereas, within specified limits, the bone undergoes an increase in cell mass. However, achieving parallel osteogenic outcomes in vitro requires higher values of strain (1–10%).^[^ ^123^, ^124^ ^]^ Stimulation of in vivo dynamics is achieved using different categories of bioreactors like spinner flasks, rotating walls, perfusion bioreactors, compression bioreactors, etc.^[^ ^125^ ^]^ To achieve uniform cell growth on scaffold and provide nutrient supply, perfusion bioreactors are utilized, which through a constant flow of the nutrient media, stimulate cells toward the mechanical shear.^[^ ^126^ ^]^ Further, inside the bioreactor, osteoblasts are subjected to mechanical stress via fluid flow (hydrostatic), gravity (static), or lever‐based (mechanostatic) stress that needs to be identical to physiological conditions to illicit anabolic cellular functions. Inside a 2‐dimensional (2D) Kilopascal (kPa) environment, all cells are subjected to identical stress values; however, bearing in mind the complicated geometry of bone, bioreactors, and other 3D models are preferred.^[^ ^7^ ^]^ Analytical techniques efficiently measure the mechanical stress in 2D models, however, for estimation of actual stress experienced by cells in a complex configuration of 3D model, it is imperative to utilize a computational approach. Lattice–Boltzmann utilized micro computed tomography (µCT) scans of human bone to obtain critical microstructural data, e.g., pore size, channel length, etc. When utilized for simulations, the data pointed to the inadequacy of flow‐induced stress alone to predict biological responses and pointed out scaffold architectural parameters to be accounted for. Computational fluid dynamics (CFD) analysis depicted 40 times higher magnitude shear stress values in CaP scaffolds than in polymeric scaffolds. Thus, CaPs are a natural choice for the construction of bone models, and the observed trend signifies the importance of utilizing CaP with higher mechanical strength for in vitro investigations.^[^ ^127^, ^128^ ^]^

Porous structures of calcium phosphate ceramics are rapidly populated by bone tissues exhibiting similar features to native bone. However, the presence of pores may coincide with a decrease in the mechanical stability of such calcium phosphate ceramics as HAp. Exploration of optimum parameters like pore size and pore volume is imperative to strike an equilibrium between effective colonialization of pores by bone tissues and mechanical resilience required for load‐bearing applications of the HAp ceramics as mechanical strength is inversely related to the pore size of the bioceramics.^[^ ^129^ ^]^

Furthermore, chemical composition and shape critically determine the strength of bioceramics. Macromolecules like collagen and bovine serum albumin and organics like urea have encouraged the formation of biomimetic nanocrystals of HAp. Moreover, carbonated HAp nanocrystals with needle‐like morphology (26 nm–1.5 µm) upon sintering above 1200 °C attained bending strength in proximity to bone (≈88 MPa).^[^ ^130^, ^131^, ^132^, ^133^, ^134^, ^135^, ^136^ ^]^ At the same time, spray‐drying of HAp alongside zirconia nanoparticles (<100 nm) and sintering of the resulting products at 1075 ± 25 °C (pressure: 4.5–17.3 MPa) resulted in enhanced toughness of the compacts obtained. Compacts (die cast ≈13 mm diameter) with porosity of 20% would withstand loads up to 1 kgF.^[^ ^137^ ^]^ Composites manufactured with synthetic polymers like polylactic acid and polycaprolactone acrylic resins (PLA‐PCL) containing HAp in nanoparticulate (<200 nm) form displayed higher mechanical strength. Incorporation of 10% HAp nanoparticles into PLA‐PCL matrix upgraded tensile strength from 16.75 to 30.68 MPa with a concomitant increase in flexural strength from 30.21 to 55.35 MPa, making them ideal for load‐bearing purposes.^[^ ^138^, ^139^ ^]^ Similarly, applications of β‐TCP have increased mainly because of its enhanced biocompatibility and biodegradability, but lower mechanical strength (3–5 MPa) compromises its utility for load‐bearing investigations in vitro. Alteration in the chemistry of β‐tricalcium phosphate with alumina followed by sintering has yielded composites with improved mechanical strength of ≈13.5 MPa.^[^ ^140^ ^]^ Similarly, post‐inclusion of silica nanoparticles (10–30 nm) in β‐TCP scaffolds enhanced compressive strength of naïve β‐TCP scaffold (3.12 ± 0.36 MPa) to higher values of 5.74 ± 0.62 MPa. In contrast, incorporation of magnesium nanoparticles (average particle size 20 nm) further elevated the compressive strength to 9.02 ± 0.55 MPa. The phenomena were attributed to the improved density of β‐TCP post doping.^[^ ^141^ ^]^

**Figure 5:**
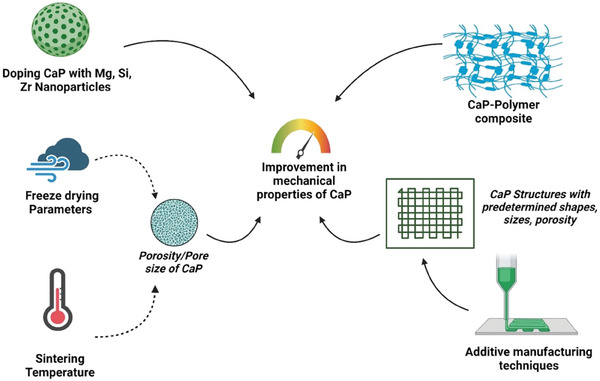
Process parameters and other factors to increase mechanical strength of CaP.

Processing techniques and associated variables also impact the mechanical properties of calcium phosphates. For instance, freeze‐drying parameters regulate the pore size of β‐TCP slurries and their mechanical properties. Freeze drying under a controlled rate (1.86 °C min^−1^) creates 4.83 µm pores with a final material compressive strength of 1.74 MPa. A constant freezing temperature of 5 °C resulted in a pore size of 2.84 µm, corresponding to a compressive strength of 2.25 MPa. Interestingly, pore dimensions were also modulated by the particle size of the CaP powders to be freeze‐dried.^[^ ^142^ ^]^ Nanosized powders create large pores (100 µm); contrarily micron‐sized powders generate smaller pore sizes below 10 µm. The gel casting approach forms materials with compressive strengths comparable to cortical bone. In contrast, freeze casting of nano‐powders laden with porogens yields constructs with mechanical properties identical to cancellous bone.^[^ ^143^, ^144^, ^145^ ^]^ Ultimately, a hierarchical structure similar to the bone would be created via biomimetic mineralization. It was shown by Liu et al. that HAp nanorods constructed on DCP microplates via bottom‐up technique when infused with gelatin, created composites with mechanical virtues similar to the cortical bone.^[^ ^146^ ^]^ Additive manufacturing is another technique providing ceramic structures with predetermined shapes, sizes, porosity, and mechanical properties. 3D printing of bioactive ceramic scaffolds with hexagonal architecture leads to enhanced mechanical properties credited to greater contact among printed subunits.^[^ ^147^ ^]^ Process variables like sintering temperature and resulting porosity regulate the mechanical features of calcium phosphates. Experimental analysis of HAp and TCP reported a decrease in porosity from 63.7% to 47.1%, with a concomitant increase in the sintering temperature from 1050 to 1250 °C. Elevation in processing temperature was also directly proportional to flexural strength and flexural modulus. Upliftment from 4.9 ± 0.06 to 38.5 ± 0.44 MPa was noted for flexural strength, whereas flexural modulus changed from 1.05 ± 0.02 to 3.08 ± 0.02 GPa with increase in temperature.^[^ ^148^ ^]^ **Figure** **5** depicts the effects of process parameters also summarizing other means to increase mechanical strength of CaP.

Compressive strength and Young's modulus for cancellous bone are 1.8–10.2 MPa and 10–2000 MPa, respectively, while corresponding values for cortical bone are 133–195 and 11.7–18.2 MPa. Such high values are achievable in ceramics only post‐reinforcement with resilient polymers like polycaprolactone, titanium particles, etc.^[^ ^149^ ^]^ It was found by Ma et al. that a Polyethylene glycol (PEG) dicalcium phosphate anhydrous‐modified CaP scaffold formed a composite with the desired mechanical and biological properties. However, casing polymer on a ceramic surface would block the osteogenic properties of the concerned material. Therefore, it is pertinent to strike the right balance between mechanical properties and the inherent biological activity of the CaP material.^[^ ^150^ ^]^

## 4 Application of Calcium Phosphates for In Vitro Bone Models

### 4.1 Application of Calcium Phosphates

Numerous reports have stated the involvement of different CaP materials in bone formation. In vivo studies with rat calvarial defects and zebrafish models advocated the presence of Amorphous Calcium Phosphate (ACP) as a precursor phase for apatite growth. Alkaline conditions inside mitochondria support ACP formation under normal physiological conditions. After that, ACP enters the gap junctions of the collagen fibers and induces apatite formation, leading to bone development.^[^ ^151^ ^]^ Contrarily, precipitation mechanisms for calcium phosphates formation studied using CaCl_2_ addition in a Na_2_HPO_4_/NaH_2_PO_4_ under constant conditions of pH (7.4) and temperature (37 °C) depict the formation of an intermediate dicalcium phosphate dihydrate (DCPD) phase as a precursor to HAp – major mineral component of bone. The observation is supported by the fact that the system is supersaturated with respect to DCPD, favoring its nucleation. Further, DCPD transits into octacalcium phosphate (OCP), which subsequently hydrolyses into HAp.^[^ ^152^ ^]^ Overall, ACP, DCPD, and OCP are all important phases to consider for the biological synthesis of HAp. **Table** **1** discusses the applications of calcium phosphates for generating in vitro bone models and their solubility product constant, (Ksp) at 37 °C.

**Table 1:** Solubility product constant, (Ksp) of calcium phosphates at 37 °C and their in vitro applications for generating bone models.

Compound	Molecular Formula	Ca/P ratio	Solubility at 37 °C, ‐lg [K sp]	Distinct In vitro Applications	Reference
Brushite (DCPD)	CaHPO_4_.2H_2_O	1	6.63	Brushite serves as source of calcium and phosphate ions to stimulate ossification process in organotypic in vitro model of bone, also providing mechanical stability	[153]
Monetite (dicalcium phosphate anhydrous‐DCPA)	CaHPO_4_	1	7.02	Monetite can induce osteogenic differentiation of hMSCs in a non‐osteogenic conditioned medium when used as scaffold forming material similar to hydroxyapatite	[154]
Octacalcium phosphate (OCP)	Ca_8_H_2_(PO_4_)_6_. 5H_2_O	1.33	95.9	OCP was reported to stimulate differentiation of Tendon stem/progenitor cells (TSPCs) in vitro and can be applied to model TSPC‐based bone–tendon junction	[155]
α‐Tricalcium phosphate (α‐TCP)	α‐Ca_3_(PO_4_)_2_	1.5	25.5	β‐TCP supports viability of human primary osteoblasts (PO), and bone marrow mesenchymal cells (BMMC) in comparison to α‐TCP α‐TCP is metastable and hydrolyses rapidly to calcium‐deficient hydroxyapatite	[156]
β‐Tricalcium phosphate (β‐TCP)	β‐Ca_3_(PO_4_)_2_	1.5	29.5		
Amorphous calcium phosphate (ACP)	Ca_x_H_y_ (PO_4_) _z_•nH_2_O	1.5	25.5–28.3	ACP coating enhances biocompatibility of hydrophobic surfaces (e.g., increased spreading of osteoblastic MC3T3‐E1 cells on polystyrene surface forming focal contacts was reported on polystyrene surface coated with ACP)	[157]
Hydroxyapatite (HA)	Ca_10_(OH)_2_(PO4)_6_	1.67	117.2	Incorporation of HA into polymeric scaffolds enhanced the mechanical strength of the scaffold in perfusion bioreactor also supporting proliferation and differentiation of MC3T3‐E1 cells	[158]

Numerous investigations have been performed in the recent past, manifesting bone functioning and its vasculature. In conventional 2D models, cells are co‐cultured on cell culture plates. Despite their high throughput nature, cultivating cells in 2D monolayers is an oversimplification of the bone structure, as the organic and inorganic matrices are absent. It would be observed that 3D models outperform the 2D ones.^[^ ^159^ ^]^ Bone requires compact cellular arrangement around mineralized deposits and microvasculature development, rendering 3D models an ideal approach. Cells seeded onto scaffolds /polymeric hydrogels are the preferred choice, as they precisely recreate the 3D pattern of the bone.^[^ ^160^ ^]^ These engineered constructs accumulate cells efficiently, enhancing the differentiation required for tissue maturation and functionality and providing mechanical strength. Being minimally complex compared to animal models, they allow control over and recording biochemical and biomechanical cues. These models resemble bone tissue's authentic ramification, including its mineralized matrix laden with human cells. Essentially, these models permit high throughput investigation and modulation in experimental parameters, simultaneously ensuring improved detection of cellular responses. An extensive review of such bone models is provided by J. Scheinpflug et al.^[^ ^161^ ^]^ Primarily, bone models are utilized to understand bone development and, secondly, to study associated pathologies, making provisions for the testing of therapeutics. Scaffolds in 3D in vitro model when cultured under static conditions such as multiwell plates tend to accumulate cells heavily at the scaffold periphery. This leads to inadequate nutrient and waste exchange in the scaffolds central region resulting in cell necrosis at scaffolds center. While hydrogels provide numerous advantages certain drawbacks have been noted like inefficient solute diffusion with constraints in applications at submicrometric scales. Further, the scattered distribution of mineral arrangement within their is structure noted only weeks after cell seeding proves their inadequacy to imitate intricate nanoscale arrangement of bone.^[^ ^162^ ^]^ For overcoming these limitations 3D in vitro bone models utilizing scaffolds and hydrogels are introduced in microfluidic devices, bioreactors etc.^[^ ^163^, ^164^ ^]^ Consequently, scaffolds/hydrogel generation forms the preliminary step toward generation of successful 3D in vitro models for bone.

**Figure 6:**
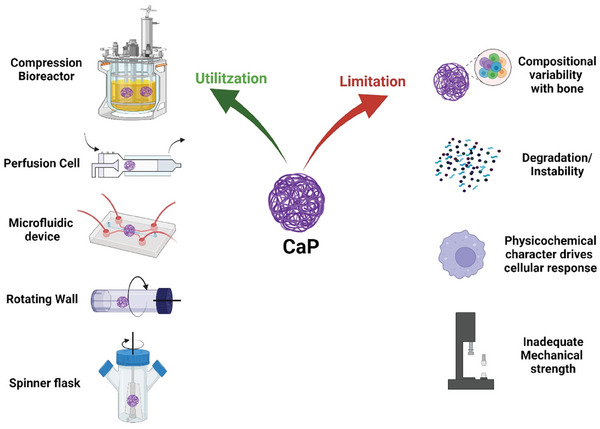
In vitro bone models that employ the scaffolds system and current limitations in utilizing CaP materials.

A major portion of the bone comprises of calcium phosphates (CaP). CaP ceramics like hydroxyapatite (HAp), β‐tricalcium phosphate (β‐TCP), and biphasic calcium phosphates (BCP) are often utilized as a first choice for bone scaffold construction mainly due to their virtue of osteoconductive and osteoinductive properties and similarity to natural bone composition and structure. These scaffolds provide biomimetic environment that supports cell adhesion proliferation and differentiation, mimicking native bone tissue. Considering their relevance, within this review article, we attempted to define the primary objective behind engineering in vitro bone models, highlighting the properties and limitations of CaPs, which, if carefully considered in a 3D scaffold, would lead to harnessing their potential to the fullest. **Table** **2** compares general properties of CaP with that of hydrogels which would aid their applicability for in vitro bone modelling, whereas; **Figure** **6** illustrates prototypical examples of in vitro bone models that employ the scaffolds system and current limitations in utilizing CaP materials into these systems.

**Table 2:** Comparative properties of Calcium phosphates and hydrogels.

	Calcium Phosphates (CaP)	Hydrogels
Advantages	Biocompatibility and Bioactivity: CaP mimic mineral component of bone promoting cell attachment, proliferation, and differentiation^[^ ^165^ ^]^ Osteoconductivity: They support new bone tissue formation^[^ ^166^ ^]^ Resorbability: Depending on the Ca/P molar ratio they are likely to gradually degrade and be replaced by new bone over time, which is beneficial for bone healing^[^ ^167^ ^]^	Flexibility and Tunability: They can be engineered to mimic wide range of tissue environment from soft to hard tissue^[^ ^168^ ^]^ Biocompatibility: Hydrogels are primarily composed of biocompatible materials that enhance cell survival^[^ ^169^ ^]^ Ease of cell encapsulation: Cells can be easily encapsulated into hydrogels during their fabrication^[^ ^170^ ^]^
Disadvantages	Brittleness: CaP are brittle and do not withstand mechanical stress, making them unsuitable for load‐bearing applications^[^ ^171^ ^]^ Complex fabrication: Generating CaP scaffolds with controlled porosity and microstructure can be technically challenging^[^ ^172^ ^]^	Degradation control: Controlling degradation rate of hydrogel‐based scaffolds is challenging which impacts its long‐term stability and in vitro performance^[^ ^173^ ^]^ Limited Osteoconductivity: Hydrogels often require functionalization with bioactive molecules promoting bone tissue formation^[^ ^174^ ^]^

### 4.2 Clinical Relevance and Patient‐Specific Modeling

Osteoporosis, osteopetrosis and rickets are bone metabolic disorders affecting the bone remodeling. This culminates either into excessive bone resorption or formation. Clinically it is imperative to maintain bone remodeling process which also necessitates generation of a suitable in vitro bone construct. 2D and 3D culture models are adopted for creating physiologically relevant bone‐like structures. They facilitate diverse tissue formation and aid in development of personalized medical therapies if patient derived cells are utilized. However, effective utilization of the different cell types for bone model development still remains a challenge owing to several shortcomings that they encompass. Heterogeneity among cell types induced in patients by disease and associated pathophysiological conditions poses a severe challenge to using generalized in vitro models.^[^ ^175^, ^176^, ^177^ ^]^ Therefore, the impersonation of patient biology in vitro calls for the induction of cells from the patient himself. Another potential shortcoming, particularly for in vitro assays using primary cells, is the biocompatibility of the material used to construct the in vitro models.^[^ ^178^ ^]^ Apart from donor variation, achieving an adequate number of osteocytes for experimental purposes is time‐consuming, for which MSC from bone marrow provides a suitable alternative. However, MSC consumes more time to differentiate.^[^ ^179^, ^180^ ^]^ Contrastingly, current research predominantly draws conclusions on cells derived from animal sources. Domaschke et al. co‐cultured mouse ST‐2 osteoblastic cells and osteoclasts differentiated from human monocytes onto mineralized collagen scaffolds containing 30% collagen and 70% hydroxyapatite crosslinked with ethyl‐carbodiimide. The study revealed that osteoclasts degraded the scaffold, facilitating extracellular matrix generation. As scaffolds were made of collagen I and hydroxyapatite with simplistic approach obtained results indicate that other constituents are inconsequential for the bone remodeling process. The two‐component scaffold enables evaluating how the extracellular matrix affects bone cell behavior. Cultivating osteoclasts and osteoblasts on the collagen mineralized with hydroxyapatite would serve as an in vitro model for bone remodeling.^[^ ^181^ ^]^ Similarly, a 3D model of bone‐tendon‐muscle connection composed of type I collagen and agarose, with a gradient in hydroxyapatite going from bone to muscle was 3D printed. Cell proliferation of osteoblast‐like cell line (MG‐63), human dermal fibroblasts (HDFs) and Sket.4U cells were studied for 21 days. It was shown that incorporation of hydroxyapatite increased the mechanical strength of collagen and its utilization in different concentrations helped to mimic the enthesis structure as well as aid the cells to grow and be more active thereby showing potential to form tissue connections. Considering obtained results such models under clinical settings would aid in regenerating injured or degenerated interfaces in patients and could be employed to study diseases, cancer, or aging at these interfaces. The developed technique could also serve as a model to study bone remodeling. The junctions among musculoskeletal tissues, varying in modulus undergo morphological and biological adaptations enabling transmission of force with minimal injury. These interfaces, i.e., tendons and ligaments have greater surface areas, contrasting protein composition and graded mineral compositions allowing smooth transfer of force.^[^ ^182^ ^]^ Despite advances in tissue engineering majority of failures in autografts and allografts harvested out of hamstring tendons arise at the implant fixation site. Limited emphasis is given to create models mimicking composition of these interfaces impeding clinical application of the engineered tissues to be translated. Primary tendon fibroblasts from murine source grown on fibrin gel anchored around brushite based cements served as a model for bone‐ligament interface. Brushite cements are more resorbable than hydroxyapatite making them ideal candidates for engineering bone ligament interfaces. Developed in vitro model was stable up to 23 days and would withstand the stress up to 21.6 ± 1.6 Kilopascal (kPa). The findings advocate potential of brushite based cements in investigating models pertaining to ligament disorders.^[^ ^183^ ^]^

Utilization of the calcium phosphate ceramic type also depends upon the projected end result. Fibrin scaffolds structurally and biochemically mimic the callus formed during healing process of a bone fracture. Therefore, if combined with calcium phosphates, fibrin‐based systems would prove useful for studying bone regeneration. Lordacheschu et al. introduced a mixture of β‐TCP and brushite as anchors for withholding a fibrin gel loaded with cells derived from the murine femur. Instead of hydroxyapatite, which resembles bone chemically, β‐TCP and brushite anchors were opted. Owing to their enhanced solubility these provided a supply of local calcium and phosphorus to aid in ossification. Further, identical matrix alignment is seen between the trabecular and cortical bone under physiological conditions providing required mechanical resistance for bone development. Analogous to this natural environment, the β‐TCP and brushite anchors used in the study provided forces to align the cell containing fibrin gel to that of natural bone. Experiment was continued throughout the year and exhibited deposition of mineralized collagenous matrix. Therefore, cell loaded fibrin gel anchored with β‐TCP and brushite serves as in vitro model to evaluate bone replacement materials (release of harmful ions from medical devices into surrounding tissues) and heterotrophic ossification process. It could also serve as a screening platform for testing drugs that either suppress or promote ossification. The model offers a way to investigate bone formation aiming to either promote or inhibit ossification in various conditions, reducing the need for animal testing in animal research.^[^ ^184^ ^]^

Cancer metastasis under clinical settings have followed the traditional “Tumor node metastasis model (TNM)” which scrutinizes factors like tumor size (T), extent of spread to lymph nodes (N), and presence of metastasis (M). However, most recent research focuses on characterizing metastatic behavior of tumor cells, and requires novel models for evaluation purposes. Metastatic breast cancer displays higher affinity toward bone and traditional in vitro models encompass limitations with respect to reproducibility and flexibility of design. Therefore, to overcome such limitations, PLA scaffolds with square and hexagonal shapes were 3D printed and functionalized with nano hydroxyapatite (nHAp). The results demonstrated that combining nHAp coating with high‐resolution 3D printed pores and specialized hexagonal shapes significantly boosted the activity of breast cancer cells and mesenchymal stem cells. Thus the developed scaffolds offer a promising bone model for studying metastatic breast cancer behavior in bone.^[^ ^185^ ^]^

Perfusion bioreactors provide the mechanical stimulation to the tested scaffolds or scaffolds combined with cells through continuous flow of the culture media. Integrating a compression system alongside or with perfusion enhances the utility of bioreactors. Scaffolds utilized for development of such perfusion bioreactors, need to withstand the mechanical stress while making provisions for the support of the cells provided. Calcium phosphate bioceramic with controlled internal structure provide exciting alternative to overcome the limitations. Macroporous bioceramic scaffolds composed of 85% TCP and 15% HAp (sintered at 1100 °C for 3 h) provide higher mechanical strength and prevent fluid flow by‐pass. Experimental results demonstrated reproducible growth and differentiation of mouse calvarial cells into osteoblasts under perfusion (flow of 2 µL min^−1^). Additionally, upregulation of C‐Fos expression under perfusion based mechanical loading highlighted the scaffolds effective mechanical stimulation of cultured cells. Hence, generated model serves as ideal prototype for evaluation of bioactive molecules under load bearing applications.^[^ ^186^ ^]^ Despite potential owing to inherent limitations, the majority of currently developed models for clinical applications do not deploy calcium phosphates. Prototypical examples summarizing of such in vitro bone models with respect to their utility are summarized in **Table** **3** below.

**Table 3:** Types of in vitro bone models with respect to application.

In vitro models to study Bone development				
Model type	Cells utilized	Study details	Study outcome	Reference
3D co‐culture and bioreactor based	Human osteoblasts and human osteoclasts from the human jawline.	Static as well as dynamic model. Static: Agarose was coated on a polystyrene 24 well plate. Dynamic: Rotary Cell Culture System (RCCS)‐4TM bioreactor (Synthecon, Inc., Houston, TX, USA).	After 21 days, the cells grown under dynamic conditions were more organized. The model would be utilized to study osteonecrosis and therapeutic agents.	[187]
3D (in vitro) hybrid	Primary human osteoblasts.	Mineralized collagen (Type 1) matrices prepared with SBF.	Osteoblasts readily differentiated with simultaneous expression of matrix metaloproteinase (MMP)‐13, integrin binding sialoprotein (IBSP), and Dentin matrix protein 1 (DMP‐1) expression. The model would be useful for studying biomineralization.	[188]

In vitro models to study Metastatic breast cancer				
Model type	Cells utilized	Study details	Study outcome	Reference
3D Bone‐on‐a‐chip	Murine calvaria preosteoblasts (MC3T3‐E1) co‐cultured with human breast cancer cell lines, MDA‐MB‐231^GFP^, and its metastatic suppressed variant MDA‐MB‐231‐BRMS1^GFP^.	PDMS structure compartmentalized with nitrocellulose membrane placed on glass support for the growth of the seeded cells.	Bone on chip supported rapid growth of 3D collagenous tissue without differentiating agents. The model is helpful in enhancing and studying the interaction of cancerous cells with bone matrix.	[189]

In vitro model to study Cleidocranial dysplasia				
Model type	Cells utilized	Study details	Study outcome	Reference
2D culture on plates	Induced pluripotent stem cell line GZHMCi003‐A sourced from umbilical cord blood mononuclear cells (UCBMCs).	Cells were seeded on Matrigel coated plates.	Deletion of exon 3 in the runx2 gene from the GZHMCi003‐A cells makes it ideal for studying pathophysiology associated with Cleidocranial dysplasia.	[190]

In vitro model to study Gaucher's disease (GD)				
Model type	Cells utilized	Study details	Study outcome	Reference
3D co‐culture model via 3D printing	Human bone marrow derived mesenchymal stem cells (hMSCs) and peripheral blood mononuclear cells (PBMCs).	Co‐cultured spheroids were aspirated and 3D printed onto the alginate surface.	Positive ALP activity and occurrence of multinucleated cells demonstrate the ability of this model in investigations involving GD pathophysiology.	[191]

In vitro Model investigating therapeutic effect				
Model type	Cells utilized	Study details	Study outcome	Reference
2D monolayer construct and 3D bio‐printed scaffolds	Bone marrow‐derived mesenchymal stem cell (BM‐MSC).	Three layered cubical configurations of a total of 27 spheroids were printed and laced onto 24 well plates. A monolayer of BM‐MSC was cultured onto the flat bottom 24 well plates.	Models were tested with four agents known to promote or impair osteogenesis. The 3D platform proved more relevant than conventional 2D for screening of drugs.	[192]

In vitro model to study Paget's disease				
Model type	Cells utilized	Study details	Study outcome	Reference
2D co‐culture	Osteoclast‐like cells from Canine bone marrow cell cultures infected with Canine Distemper Virus (CDV).	Cells were grown on 8 well tissue culture slides.	CDV infection induces interleukin‐6 and c‐Fos mRNA expression in cultured cells, suggesting its involvement in the pathogenesis of the disease. A model can be useful for assessing the disease progression in detail.	[193]

In vitro model for studying malignant bone tumour's (MBT)				
Model type	Cells utilized	Study details	Study outcome	Reference
Bioreactor model	Co‐culture of osteogenically enhanced human mesenchymal stem cells (OEhMSCs) and MOS‐J cells.	3D in vitro model to evaluate/study bone–tumor interactions in a Rotating wall vessel bioreactor (RWV).	The tumor cells stop osteogenesis by disrupting canonical Wnt (cWnt) function in OEhMSCs. The model provides a better understanding of the pathogenesis of MBT.	[194]

In vitro model for studying Prostrate cancer (PC) bone metastasis				
Model type	Cells utilized	Study details	Study outcome	Reference
3D co‐culture model via 3D printing	Human osteoblast–derived bone‐like microtissue (hOBMT) and metastatic prostate cancer cell lines (LNCaP, C4‐2B, and PC3 cells).	Porous polycaprolactone scaffolds prepared by melt electrowriting were coated with CaP and then seeded with cells.	Sclerostin was expressed in 3D constructs that activated the nuclear factor κ B/osteoprotegerin (RANK/OPG) pathway of prostate cancer cells, affecting the nuclear factor κ B/osteoprotegerin (RANK/OPG) pathway of prostate cancer cells, affecting the nuclear factor κ B/osteoprotegerin (RANK/OPG) pathway of prostate cancer cells affecting metastasis.	[195]

In vitro model for studying Rheumatoid Arthritis (RA)				
Model type	Cells utilized	Study details	Study outcome	Reference
2D cell culture model	Cells from synovial tissues of rheumatoid arthritis and osteoarthritis patients.	Cells were grown on eight well chamber plates.	An in vitro replica of the rheumatoid synovium depicting cellular growth, activation, and polarization with bone resorption was generated.	[196]

In vitro model for studying Myeloma Bone Disease (MBD)				
Model type	Cells utilized	Study details	Study outcome	Reference
3D co‐culture (Bone organoid)	Bone marrow stromal cell–derived osteoblasts (OBs) and bone marrow macrophage (BMM)–derived osteoclasts (OCs) and human multiple myeloma (MM)–derived plasmacytoma cells.	Multiple myeloma cells were introduced into the organoid culture of displaying endosteal‐like extracellular matrix (ECM), abundant alkaline phosphatase, calcium, and markers of osteoblastic gene expression.	Multiple myeloma cells reduced HAp content and expression of osteoblastic genes. Restoration was achieved with therapeutic intervention employing immunomodulation and bisphosphonate therapy.	[197]

## 5 Challenges in Utilization of Calcium Phosphates for In Vitro Bone Models

Calcium phosphates induce de novo bone formation via numerous pathways. First, they release Ca^2+^ and PO_4_^3−^ ions into the surrounding biological milieu, attracting osteogenesis.^[^ ^198^ ^]^ Second, a momentous event denoting the bioactivity of CaP ceramics is the deposition of carbonated apatite onto their surface.^[^ ^198^, ^199^ ^]^ This event further draws the adsorption of various growth factors like BMP and VEGF. It is imperative to note that CaP casts both an osteoconductive as well as osteoinductive role. Osteoconduction is the ability of materials to trigger the differentiation of bone‐forming cells into osteogenic cells, whereas osteoinduction refers to the migration and recruitment of cells to the site. In vitro models to recreate individual bone firmly depend upon CaP ceramics and scaffold‐based approaches laden with osteoprogenitor cells.^[^ ^41^, ^198^ ^]^ Despite the advantages of calcium phosphate materials in mimicking the mineral phase of natural bone, their underutilization in addressing the current challenges in in vitro bone modeling can be attributed to several factors:

### 5.1 Compositional Variability

Bioapatite the mineral component of bone is chemically complex, non‐stoichiometric and rich in defects. A more accurate formula for it would be (Ca, Mg, Na)_10‐x_[(PO4)_6‐x_(CO_3_) _x_] (OH)_2‐x,_ where minor components like magnesium, sodium, and carbonates compensates for charge imbalances. It constitutes 65–70% of bone while the rest is comprised of collagen, water, proteins and other small organic molecules. Bioapatite contains 8% by weight of carbonate.^[^ ^200^ ^]^ Furthermore, the levels of trace elements like manganese, zinc, copper and iron regulate bone formation and transformation.^[^ ^201^ ^]^ However, incorporation of such bioactive ions is bound to enhance the solubility of formed apatite crystals in synthetic biological fluids or in cell culture media. Therefore, systematic efforts are must to produce stable ion doped apatite crystals and apply those in in vitro bone models.^[^ ^202^ ^]^ Additionally, different organic groups like acetate, citrate, ascorbate, glutamate, and itaconate interact with inorganic materials and play pivotal role in bone regeneration. Bound citrate is reported across fish, avian, and mammalian bone, indicating its critical role in interfering with crystal thickening and stabilization of the apatite nanocrystals in bone.^[^ ^203^, ^204^ ^]^ Whereas, macromolecules such as collagen, and dentin matrix protein 1 (DMP1) are implicated in nucleation, crystallization, aggregation, and phase transformation of amorphous calcium phosphates to thermodynamically stable hydroxy apatite.^[^ ^205^ ^]^ These components are decisive in biomineralization and therefore focused approach is required to study relationships of small organic molecules and macromolecules in models describing bone formation. Currently, calcium phosphate material utilized into in vitro bone models lack compositional similarity to that of bioapatite and therefore, are deficient in replicating exact bone structure. While calcium phosphate materials provide a mineralized scaffold, they alone may not fully replicate the complex biochemical and cellular interactions present in living bone tissue. Hence researchers must work on advanced strategies to deploy these materials and in order to better mimic bone chemically.

### 5.2 Degradation/Instability

In view of in vitro models’ application of calcium phosphates is challenging. Thermodynamically most calcium phosphate ceramics exhibit certain solubility in aqueous media as noted from their low solubility products. Controlling the degradation rate of calcium phosphate materials is crucial, but it may not be easily achievable with certain compositions.^[^ ^206^ ^]^ Researchers may explore alternative materials with tunable degradation profiles or adopt sophisticated approaches like surface modifications to control the release of ions. The quest for more versatile degradation kinetics drives the exploration of different biomaterial options.

Acidic calcium phosphates like DCPA and OCP dissolve readily at neutral pH whereas α‐TCP and amorphous calcium phosphates are highly soluble under similar conditions. HAp is relatively stable under physiological conditions however, its stability diminishes with increased nonstoichiometry leading to calcium deficiency and impurities like calcium carbonate. Comparative resorption of β‐TCP is faster than HAp; however, it starts to dissolve at pH < 6. Solubility attributes determine the relative stability of calcium phosphate ceramics and their applicability for in vitro models.^[^ ^9^, ^207^ ^]^ OCP is the only nonceramic calcium phosphate studied extensively for bone regeneration purposes. Enhanced proliferation of bone marrow stromal ST‐2 cells and primary calvarial osteoblasts cells from murine origin was reported in vitro on cell culture dishes coated with OCP compared to calcium deficient HAp. Additionally, when comparing OCP with calcium‐deficient HAp in a rat calvarial bone defect model, there was an observed increase in bone formation when identical amounts (15 mg) of the powders were applied to the defect site.^[^ ^208^ ^]^ Titanium implants (5 mm in diameter and 10 mm in length) coated with OCP (≈30 µm thickness) instigated bone formation in vivo at the ectopic site, as examined in the goat model. Therefore, OCP is known to possess both osteoinductive and osteoconductive behavior. However, OCP readily hydrolyses into HAp under aqueous conditions and reacts differently to various in vitro model solutions (pH 7.2–7.4). The composition of different in vitro model solutions (e.g., normal saline, Dulbecco's phosphate‐buffered saline (DPBS), supersaturated calcification solution (SCS)) alters the extent of OCP hydrolysis, casting profound impact on its surface reactivity, and biological performance. While normal saline causes rapid dissolution of OCP crystal structure, the opposite action is mediated by DPBS, which maintains constant OCP structure by replenishing phosphate ions. Additionally, in SCS, HAp formation occurs on OCP crystal surface.^[^ ^209^ ^]^ When exposed to a diverse range of in vitro solutions, these variations exhibited by OCP constrain its utility for constructing the in vitro bone models. Similarly, the quick resorption properties of α‐TCP limit their application in the domain of new bone formation and, consequently, for the generation of in vitro bone models.^[^ ^210^ ^]^ On the other hand, HAp exhibits the most stable calcium phosphate phase at neutral pH, followed by β‐TCP, OCP, Dicalcium Phosphate (DCP), and DCPD.^[^ ^211^ ^]^

### 5.3 Mechanical Properties

**Figure 7:**
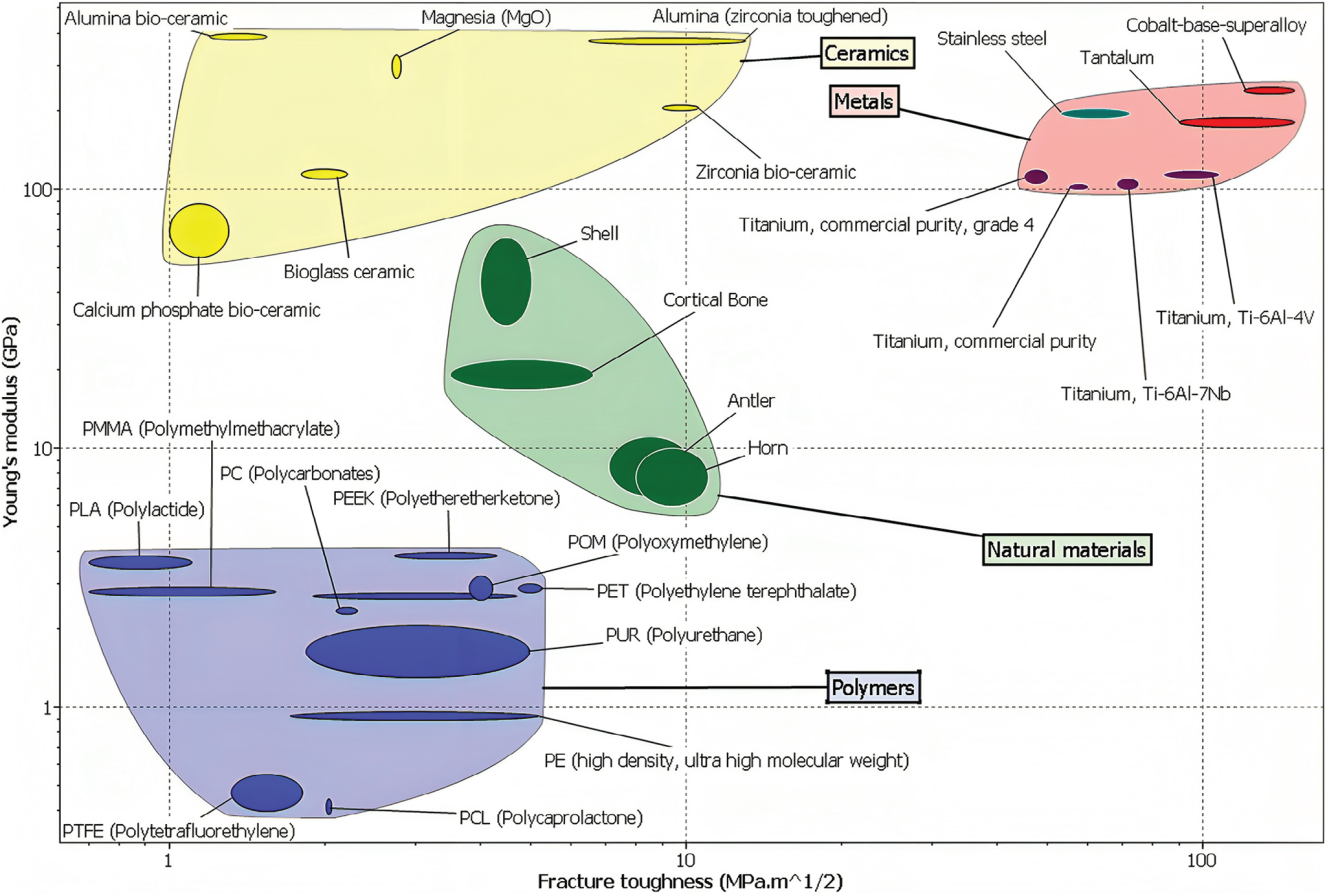
Comparative mechanical strengths of various ceramics, their composites, and human bone.^[^ ^171^ ^]^

Achieving the precise mechanical properties of bone solely with calcium phosphate materials can be challenging. Various calcium phosphate materials, such as TCP, HAp, and their combination – BCP, have been thoroughly investigated as ideal candidates for reconstructing bone.^[^ ^212^ ^]^ The ability to provide static mechanical properties (e.g., stiffness, hardness), promote osteointegration, and possess chemistry similar to that of original bone are major factors advocating their utilization. Nonetheless, their toughness and resistance to fatigue still constitute major areas for improvement, especially when it comes to load‐bearing applications, as most of the CaP ceramics are brittle with lower fracture toughness values compared to native bone.^[^ ^213^ ^]^ Compact bone sourced from cadaver tibiae (age 36–96 years) displays an average fracture toughness of 3.12 ± 1.21 MP m^−1^, whereas CaP ceramics display lower values of ≈1 MP m^−1^ as depicted in **Figure** **7**.^[^ ^171^, ^214^ ^]^

Mechanical stimulation is crucial for bone metabolism and is directly linked with bone remodeling.^[^ ^215^ ^]^ Therefore, it is of paramount importance to choose an appropriate CaP that would sustain the experimentally induced alteration in mechanical stress while making provision for the growth of cellular components.

### 5.4 Cellular Response

Osteoconduction facilitates bone growth along a surface or within a scaffold, promoting the formation of new bone tissue. At the same time, osteoinduction stimulates undifferentiated inducible osteoprogenitor cells that have not yet committed to osteogenic lineage, leading to the formation of osteoprogenitor cells. Calcium phosphate biomaterials and ceramics facilitate the development of new bone and establish a strong connection with the newly formed bone; therefore, by definition, are termed osteoconductive materials. Conversely, while calcium phosphate ceramics lack inherent osteoinductive qualities, it is possible to impart osteoinductive properties to CaP materials via modulation of their physicochemical characteristics. Surface geometries like the presence of concave/convex surfaces, micropores (≈1 µm), surface area (0.7–1.6 m^2^ g^−1^), and surface chemistry, i.e., the presence of adsorbed proteins like BSA are important factors imparting osteoinductivity to the calcium phosphate ceramics.^[^ ^52^, ^59^, ^216^, ^217^ ^]^ Osteoinduction of CaP ceramics is proven for α‐TCP, β‐TCP, BCP, α‐pyrophosphate, and β‐pyrophosphate in canine, murine, and baboon models. Despite the utility of HAp, β‐TCP, and BCP, a lack of clarity exists on the comparative osteoconductive/osteoinductive potential for these materials, CaP materials possess osteoinduction properties of their own that are governed by their chemistry and also upon the animal model into which it is introduced, like murine, rabbit, canine, etc.^[^ ^218^ ^]^ Attaining reproducibility and uniformity in the bone in vitro model is, therefore, highly dependent on its cellular interaction linked with the surface and physicochemical properties of CaP material being investigated. Calcium phosphate materials provide a suitable substrate for cell attachment and mineralization, but the in vitro cellular response may not fully mirror the in vivo scenario. Hence, researchers need to predefine and select surface and physicochemical properties of CaP material and the integration of their 3D scaffolds in, organoids, or bioreactors to enhance cellular environment and better simulate the in vivo conditions.

In summary, while calcium phosphate materials remain essential in in vitro bone modeling, the current challenges necessitate a multidisciplinary approach. Explorations are underway for combinations of biomaterials, advanced fabrication techniques, and a deeper understanding of bone biology to create more sophisticated and physiologically relevant in vitro models. The underutilization of calcium phosphates may stem from a recognition that addressing these challenges requires a broader and more integrated strategy in biomaterials research. Bone models are expected to precisely duplicate the progressive involvement of osteoprogenitors generating bone matrix in the form of osteocytes and modulate bone function from within. Therefore, owing to predictable behaviour among various calcium phosphates – HAp, β‐TCP, and BCP are mostly investigated for developing in vitro bone models.

## 6 Future Directions

**Figure 8:**
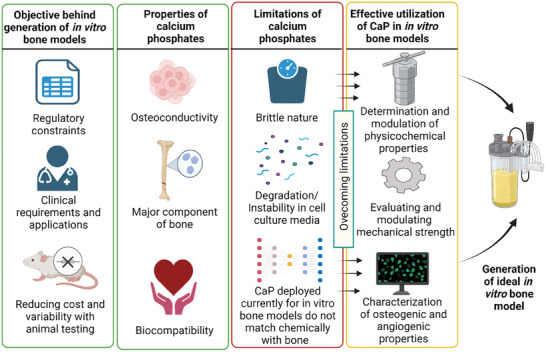
Schematic representation describing the objective behind the development of in vitro bone models and harnessing the potential of calcium phosphates to the fullest.

An optimal balance between resorption of CaP and bone tissue generation is imperative for exerting precise control over in vitro bone formation. The physicochemical character of CaPs governs both the growth and proliferation of cells (osteoblasts, osteoclasts, and endothelial cells), imparting osteoconductive and osteoinductive nature of used material. Likewise, modulation in the ability of calcium phosphates to enhance angiogenic potential and provide mechanical strength to the in vitro construct could be attained by moderating its chemical composition and topology, utilizing polymers, and appropriate processing techniques. Therefore, approaches discussed in Section 4 and as illustrated in **Figure** **8** are to be considered for the uplifting potential of CaP that would ensure the construction of efficient in vitro bone models. Thus, a three‐step screening procedure that we offer to identify and optimize suitable calcium phosphate candidates is:

(i) Appropriate characterization of osteogenic (employing computed tomography, immunofluorescence assay, alkaline phosphatase activity, etc.) and angiogenic capabilities (using ELISA, PCR) of CaPs in a construct to stimulate angiogenesis should be performed.^[^ ^66^ ^]^ This would ease the formation of new bone and ensure nutrient supply.
(ii) Determination and modulation of physicochemical properties of calcium phosphates: the ability of CaP like brushite, hydroxyapatite, tricalcium phosphate, etc., to undergo resorption depends on their respective Ksp values, which are directly linked to their biological responses.^[^ ^219^ ^]^ CaP materials exert varying biological outcomes concerning being osteoconductive or osteoinductive, depending upon their chemical composition and topological features. Simultaneous promotion of angiogenesis and osteogenesis would also be achieved with the formation of microgroved surface scaffolds containing biocompatible polymers like collagen and calcium phosphate nanoparticle scaffolds with 290 µm concave and 352 µm convex parallel grooves exhibit concomitant promotion of angiogenesis and osteogenesis.^[^ ^220^ ^]^
(iii) Evaluation of the mechanical strength of calcium phosphate and its modification: Compressive strengths of cortical and cancellous bone are 130–180 and 4–12 MPa, respectively.^[^ ^221^ ^]^ There is still a limited amount of literature available estimating the comparative compressive strengths of CaP materials as these are not usually synthesized with uniform processing parameters, resulting in identical characteristics pertaining to dimension, porosity, chemical composition, surface topology, and internal geometries. However, the brittle nature of CaP is well documented, which could be overcome by employing CaP in nanoparticulate form, casting its composite, managing porosity, and doping with ceramics like zirconia and alumina. Significant impact would also be obtained by changes in the processing parameters like sintering temperature, rate of freeze drying, and utilization of techniques like gel casting. Additionally, the incorporation of nanoparticles and the inclusion of polymer composites within the matrix of the in vitro construct should be practiced.

## 7 Conclusion

Calcium phosphate's (CaPs) physiological relevance plays a crucial role in the generation of in vitro bone. Despite this importance, the development of in vitro bone models often overlooks calcium phosphates and their scaffolds. This oversight is primarily due to the interdisciplinary focus on other biomaterials, which shapes the evolving landscape of the field. As a result, the potential of CaPs remains underutilized, and several challenges need to be addressed for further advancement.

To fully harness the benefits of CaPs, comprehensive analyses of their chemical composition and structural geometries are essential. Understanding these interactions within bone model systems requires a blend of bone tissue engineering and materials science principles. By integrating these disciplines, we can develop in vitro bone models that meet high‐quality standards, paving the way for significant progress in the field.

## Conflict of Interest

The authors declare no conflict of interest.
